# Genomic Signatures of Historical Allopatry and Ecological Divergence in an Island Lizard

**DOI:** 10.1093/gbe/evw268

**Published:** 2016-11-09

**Authors:** Richard P. Brown, Steve Paterson, Judith Risse

**Affiliations:** ^1^School of Natural Sciences & Psychology, Liverpool John Moores University, Liverpool, United Kingdom; ^2^Department of Integrative Biology, University of Liverpool, Liverpool, United Kingdom; ^3^Department of Evolutionary Biology, University of Edinburgh, Edinburgh, United Kingdom

**Keywords:** allopatry, ecological divergence, evolutionary genomics, RAD-seq, selection, speciation

## Abstract

Geographical variation among contiguous populations is frequently attributed to ecological divergence or historical isolation followed by secondary contact. Distinguishing between these effects is key to studies of incipient speciation and could be revealed by different genomic signatures. We used RAD-seq analyses to examine morphologically divergent populations of the endemic lizard (*Gallotia galloti*) from the volcanic island of Tenerife. Previous analyses have suggested ecological and historical causes to explain the morphological diversity. Analyses of 276,483 single nucleotide polymorphisms (SNPs) from >20 Mbp of the genome revealed one genetically divergent population from Anaga, a region associated with divergent mtDNA lineages in other Tenerife endemics. This population also has a high number of private alleles, and its divergence can be explained by historical isolation. Bayesian outlier analyses identified a small proportion of SNPs as candidates for selection (0.04%) which were strongly differentiated between xeric and mesic habitat types. Individual testing for specific xeric–mesic selection using an alternative approach also supported ecological divergence in a similarly small proportion of SNPs. The study indicates the roles of both historical isolation and ecological divergence in shaping genomic diversity in *G. galloti*. However, north–south morphological divergence appears solely associated with the latter and likely involves a relatively small proportion of the genome.

## Introduction

Knowledge of the processes that drive population divergence and ultimately speciation are essential to understanding the generation of biological diversity. MtDNA based phylogeography (Avise et al. 1987) developed rapidly during the 1990s and provided strong evidence of historical dispersal or vicariance and in turn led to reduced emphasis on the impact of selection. More recently, there have been clear indications that divergence with gene flow due to ecological differences has been a major diversifying force and led to the evolution of different phenotypes (Rundle and Nosil 2005; Hohenlohe et al. 2010; Ng et al. 2016).

A key question for many empirical studies of contiguous natural populations is to distinguish between ecological and historical divergence in allopatry (primarily through drift) followed by secondary contact (Thorpe 1976; Endler 1977). This should be achievable through genomic analyses. Under local adaptation, it is expected that relatively few loci would be divergent between habitats, representing solely loci under selection and neighbouring regions in linkage disequilibrium (i.e., genomic islands). Under allopatric divergence, all loci have been exposed to historical isolation. Although some ancestral and novel mutations will have been subject to selection during this period, the majority of loci will have been subject to drift alone (Nosil et al. 2009). Nevertheless, both neutral sites and those under selection should show the same geographical patterns of population divergence.

Genome-wide screening for polymorphisms in model and non-model organisms has already had considerable impact within evolutionary biology, for example, Hohenlohe et al. (2010), Nosil et al. (2012) and Guo et al. (2015). Recent next-generation sequencing approaches such as Restriction-site Associated DNA sequencing (RAD-seq) may provide enough genomic information to make it a reasonably economical alternative to whole genome sequencing for population studies involving a large number of individuals. An additional advantage of RAD-seq is that is applicable to non-model species. While analyses of biogeographical patterns attributable to historical allopatry have traditionally analyzed mtDNA and a small number of nuclear markers, RAD-seq vastly increases the proportion of the genome that is studied, allowing more detailed and robust assessments of the processes underlying population divergence as well as helping identify genomic regions of interest.

The lizard *Gallotia galloti* is abundant across the Canary Island of Tenerife (28.27N, 16.64W), from sea level to over 3,000 m. It shows pronounced geographical variation in morphology across this relatively small island (2,034 km^2^). The height of Tenerife (3,718 m) and the year-round trade winds combine to cause cloud formation on the north facing slopes, while the south-facing slopes are frequently cloudless. This leads to a dichotomous north/south contrast in vegetation which is illustrated by the mesic vegetation found at ∼400 to 1,200 m on the north facing slopes, originally comprising dense laurisilva cloud forest, tree heath and wax myrtle, and the very xeric conditions in the south which is characterised by arid-adapted *Euphorbia* plant communities (Thorpe and Brown 1989a). The primary pattern of geographic variation in the dorsal colour of adult males closely matches these xeric/mesic components (Thorpe and Brown 1989a, 1989b) as does adult male body size (Thorpe and Brown 1991). Different selection pressures between these habitats appear to be the most likely explanation. Higher population *F*_ST_ values between the N/S colour morphs have been described using microsatellite loci, leading to inferences of decreased gene flow and incipient speciation (Thorpe and Richard 2001). However, other components of the morphology, such as scalation, show different geographical patterns (Thorpe and Baez 1987; Brown et al. 2006) indicating that the origins of the morphological diversity are complex.

**Fig. 1.—:**
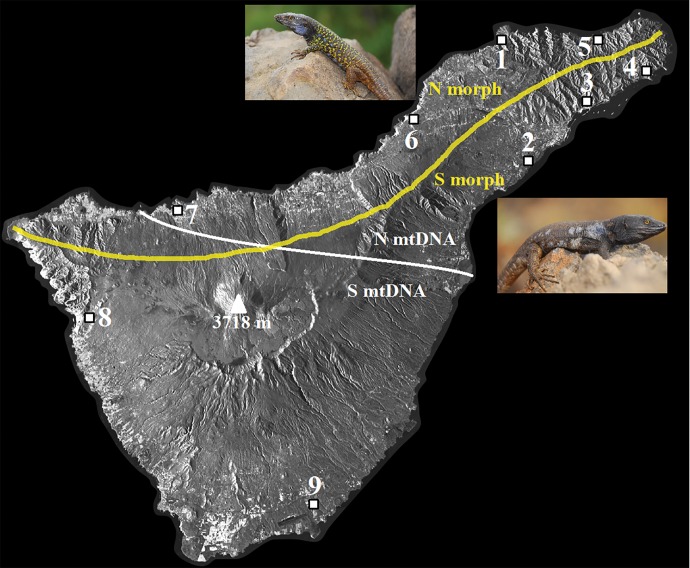
Morphological variation and mtDNA distribution in *Gallotia galloti* on Tenerife. The mid-point of the range of population variation in adult male colour pattern (adapted from Thorpe and Brown 1989a) (yellow line), the approximate distributions of the main two mtDNA lineages (white line), and the sample sites for the present study are shown on a satellite image of Tenerife (© DLR e.V. 2010 and © Airbus DS Geo GmbH 2010).

Full identification of the causes of the within-island variation is also complicated by the presence of two mtDNA lineages in *G. galloti* that diverged around 0.8 Ma (Brown et al. 2006). They show clear geographical structuring between NE and Centre/SW populations (fig. 1). The lineages are thought to have originated during population fragmentation (Thorpe et al. 1996; Brown et al. 2006). Irrespective of the actual mechanism, divergence in allopatry provides an alternative to the hypothesis of in situ differences in selection pressures. However, this structuring is discordant with the pattern of morphological variation described above and led to the suggestion that the geographical patterns of mtDNA and morphological variation have independent causes (Thorpe et al. 1996; Thorpe and Richard 2001).

The purpose of this work was to examine the utility of the RAD-seq approach in distinguishing between the hypotheses of divergent selection between habitats and historical isolation in explaining the habitat-correlated morphological variation in *G. galloti*.

## Materials and Methods

*G. galloti* were obtained from nine sites across Tenerife (fig. 1). From here on, these will be referred to as the “sampled populations” (to avoid confusion; “sites” will only be used to refer to genome positions). Sampling was designed to include proximate pairs of northern and southern slopes populations as well as to cover areas occupied by both mtDNA lineages. Tissue samples were obtained from all individuals and stored in DNAgard^®^. All lizards were released unharmed at the point of capture.

Whole genomic DNA was extracted using a Qiagen DNeasy blood and tissue kit. RAD-seq was carried out by Edinburgh Genomics on 1 μg of DNA from each of 148 samples, representing 135 individuals (2/3 replicate samples were analyzed for 12 individuals). SbfI RAD libraries were prepared and digested DNA was then ligated to P1 adapters with individual barcodes; see Davey et al. (2013). Fragments were sonically sheared to size range 300–600 bp. P2 adapters were ligated to sheared ends. Libraries were PCR amplified and sequenced on an Illumina HiSeq 2000 flowcell. This protocol provides two sets of reads at each RAD site: read 1 (117 bp) extends either side of the site, while read 2 sequences are more loosely distributed extending up to ∼700 bp from the site (Davey et al. 2013). Raw data have been submitted to the European Nucleotide Archive (Bioproject ERP015030; to be released).

The sequences were analyzed following the RADmapper approach (https://github.com/tcezard/RADmapper) based on the Stacks RAD pipeline (Catchen et al. 2013). More specifically, individual samples were demultiplexed with process_radtags allowing one mismatch in the restriction enzyme recognition site. Read 1 sequences from each sample were individually clustered using ustacks with parameters –M 3 –N 5. The resulting stacks were merged across samples using cstacks with default parameters. The final merged stacks were filtered to remove potential erroneous stacks by only retaining the ones supported by 90 or more individuals. To extend the usable sequence for SNP calling, read 2 sequences were assembled for each read 1 stack using idba_ud (Peng et al. 2012). To allow accurate assembly, we randomly selected one individual from each population and subsampled the number of reads per stack to a maximum of 1,000. The resulting read 2 assembly was then merged with the read 1 stack where possible, otherwise the sequences were concatenated. Variants were called using samtools/bcftools v0.1.18 (Li et al. 2009; Li 2011). Potential problems that can arise when assembling sequences containing indels were checked using USTACKS (Edgar 2010).

Replicate samples were removed prior to genomic analyses. Six non-replicated individuals (from five populations) were also removed because fewer than 50% of reads mapped to the assembled tags, leaving 127 individuals in the final analysis. Sample sizes for populations 1–9 were: 17, 16, 14, 17, 16, 16, 12, 16, 5, respectively. This provided an initial set of SNPs which was filtered to leave a smaller group of very reliable SNPs. The following filtering criteria were applied: (i) the proportion of individuals containing the minor allele was greater than 0.01, (ii) the SNP had been characterised in >80% of individuals, (iii) a minimum genome quality score of 50.

Multivariate analyses of SNP data were carried out using adegenet (ver. 2.0.0; Jombart 2008) and associated packages for R. A Principal Components Analysis (PCA) was used to examine the overall differentiation among individuals irrespective of population of origin. Individual alleles were coded as single 0, 1, or 2 state variables (corresponding to the allele being absent, present as heterozygote or present as homozygote, respectively). Individual scores for orthogonal Principal Components (PCs) that were found to have greatest predictive power were retained and input into a Discriminant Function Analysis (DFA). Predictive power was assessed by cross-validation. Individuals were grouped by population for the DFA, which maximizes among-group relative to within-group variation. This analysis is referred to as a Discriminant Analysis of Principal Components (DAPC) and is preferred to a standard DFA of raw allele data because it helps overcome (1) the requirement that the number of variables must be less than the number of individuals and (2) the problem of high variable colinearity, which should be particularly acute for SNP data because each locus is represented by more than one variable and because of linkage disequilibrium between SNPS within RAD sites, see Jombart et al. (2010).

Bayescan (Foll and Gaggiotti 2008) implements a Bayesian reversible jump MCMC algorithm for detecting outliers. It was used to detect candidate loci under selection. The method calculates population-locus *F*_ST_ values, which comprise a genome-wide (i.e., all SNP sites) population specific component, and a population-wide within-SNP site component. Positive selection is inferred when the population-wide SNP-site component explains a proportion of the population-locus *F*_ST_ (in addition to the population-specific component). Purifying or balancing selection is inferred if the population-locus *F*_ST_ is less than the population-specific component. The prior odds specified for the ratio of neutral: selected sites reflected the belief that neutral sites might be around 1,000 times more frequent than sites under selection; see Lotterhos and Whitlock (2014). The sensitivity of the analysis to this prior was investigated by use of alternative specifications (100:1, 10,000:1). The MCMC chain was run for 50,000 burn-in steps followed by 50,000 steps that were sampled to estimate the posterior. Significance was determined under a false discovery rate of 0.05. RAD sequences that contained SNPs identified as candidates for selection were entered into a BLASTn search, with an “Expect” threshold of 10^−04^.

The Bayesian MCMC program Bayenv 2 (Coop et al. 2010) was used to detect habitat-related selection on individual SNPs through associations with allele frequencies. Local divergence can be subject to population structuring effects, and so a variance–covariance matrix (of mostly neutral loci) provides a null model of between-population variation in allele frequencies. In Bayenv 2, this is tested against an alternative model of ecological variation. The between-population variance–covariance matrix was estimated from a sample of 10,000 randomly selected SNPs that excluded SNPs potentially under selection (as detected by Bayescan). It was obtained by running the MCMC chain for 100,000 iterations and selecting the posterior variance–covariance matrix from the final iteration as the most reliable estimate (repeated three times to ensure that different runs led to the same matrix). Each SNP was individually tested against a two-state environmental variable (standardised to a mean of zero and standard deviation of one) that reflected habitat differences between the mesic north-facing slopes and the more xeric southern slopes; see Thorpe and Brown (1989a). Bayenv 2 calculates a Bayes factor for each SNP, which is the ratio of two posterior probabilities: that for the data under the alternative model (xeric–mesic habitats) over that for the data under the null model (Coop et al. 2010). Bayes factors >10 are generally considered as providing strong evidence for the hypothesis of interest (Kass and Raftery 1995); however, Bayenv 2 analyses do not fully control for population structure and therefore strictly only provide relative indices of possible selection intensity for a particular environmental variable (Coop et al. 2010). The MCMC chain was run for 80,000 iterations on each SNP, with a sampling interval of 500.

## Results

**Fig. 2.—:**
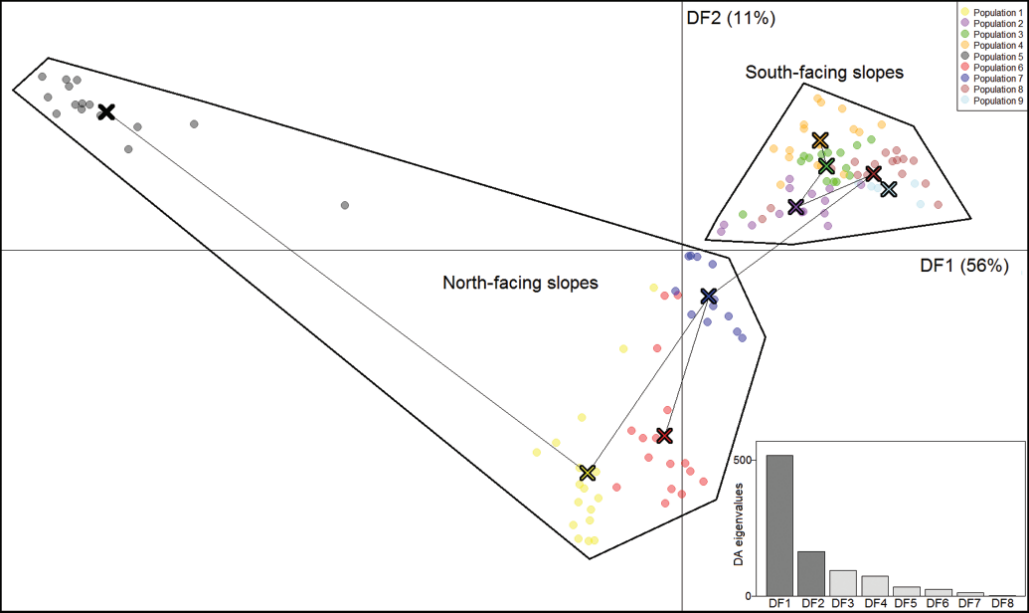
Discriminant Analysis of Principal Components, using all SNPs. Individual scores on the first two discriminant functions from the Discriminant Analysis of Principal Components of all 267,483 SNPs (axes are marked at respective origins). Lines between populations are from a minimum spanning tree between population centres (marked by X). The eight eigenvalues are given in the subplot.

**Fig. 3.—:**
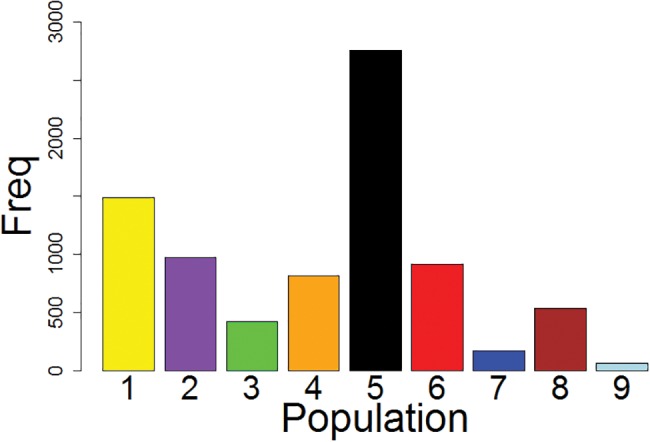
Bar chart showing numbers of private alleles in each sample relative to all other sampled populations.

A total of 276,483 SNPs from 53,565 RAD sites were retained for analysis after filtering. This represented a coverage of 20,545,118 bp of sequence from across the genome. The dimensionality of the SNPs could not be substantially reduced by PCA: successive PCs explained gradually smaller proportions of the inter-individual variance. However, the first two PCs (representing only 2.8% of the total variation among individuals) indicated a clear pattern of differentiation among sampled populations (not shown). The analysis grouped individuals from sampled populations on the south-facing slopes into one tight cluster. Individuals from most north-facing slopes formed a separate, more diffuse, cluster with individuals from northern site 5 being divergent on PC1. Cross-validation determined that use of the first 20 PCs for the DAPC was optimal. Although the 20 PCs portrayed only 20.2% of the variance, it gave the highest predictive success (92%) and the lowest root mean squared error (11%). It was more successful in reducing the dimensionality of the data than the PCA, with the first two discriminant functions representing 73.8% of the total variation (fig. 2). Nonetheless, the general patterns of differentiation were similar between the DAPC and the PCA, although the DAPC provided greater resolution. DF1 (44.4% of the variation) discriminated between three sites from the north-facing (1, 6, 7) and south-facing slopes (2, 3, 4, 8, 9) although northern site 5 was clearly divergent from all other sites (fig. 2). Calculations of numbers of private alleles within each population (relative to all other populations) indicated that population 5 contains the greatest number of unique alleles, while population 9 contains the fewest (fig. 3).

**Fig. 4.—:**
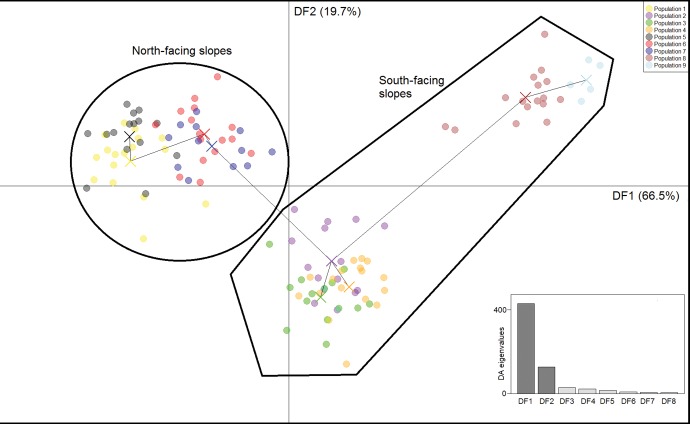
Discriminant Analysis of Principal Components for SNPs under selection. SNPs Individual scores on the first two discriminant functions from the Discriminant Analysis of Principal Components of the 103 SNPs identified as candidates for selection by Bayescan. Lines between populations are from a minimum spanning tree between population centres (marked with X). The eight eigenvalues from the Discriminant Analysis (ordered DF1–DF8) are given in the subplot.

Bayescan analyses were sensitive to the prior ratio of sites under selection to neutral sites. The number of outlying SNPs that were potentially under selection was 151 for prior odds of 1:100, 103 (from 84 RAD sites) under prior odds of 1:1,000, and 78 under prior odds of 1:10,000. Irrespective of these differences, the key finding is that only a very small proportion of the 276,483 analyzed SNPs show any evidence of selection. We consider only the intermediate 1:1,000 analysis as this provided the closest match between the prior and the posterior ratios. It was found that most (84%) of the 103 candidate SNPs identified by this analysis had very high PC1 variable loadings in the previously-described DAPC of all 276,483 SNPs (they were within the top 1% of variable loadings of individual SNPs). This indicated that many of the SNPs potentially under selection contributed significantly to the main component of inter-individual variance. A DAPC was computed on the first 40 PCs (established through cross-validation) derived from these 103 SNPs, representing 92.5% of the total variation. This provided clear clustering of the populations from the north-slopes, indicating habitat and morphology-related divergence at these 103 positions that appear to be under selection. It also indicated mutually exclusive geographical site groupings within sites from the southern slopes: sites 2–4 in the NE of the island were divergent from the two southern sites in the W and S of the island (sites 7, 8) (fig. 4). Interestingly, patterns in these SNPs differed from generalised patterns across all SNPs, inasmuch as individuals from population 5 were not divergent; they were grouped with the other morphologically similar individuals/populations from the north-facing slopes.

Only 34 of the 84 RAD sites that contained outlying SNPs produced hits on BLASTn. Excluding hits with <30% query coverage, four sequences corresponded to microsatellite flanking regions, one corresponded to a retrotransposon reverse transcriptase and another one to a SINE region (all in Lacertid lizards). A further 12 sequences with ≥30% coverage aligned with protein product predictions from the genomes of the lizard *Anolis carolinensis* (five hits: a spectrin repeat, scavenger receptor, protocadherin gene alpha subcluster, avian erythroblastic leukemia viral oncogene homolog 4, one uncharacterised protein), the snake *Python bivittatus* (three hits: APOBEC1 complementation factor, beta-site amyloid beta (A4) precursor protein (APP)-cleaving enzyme 2, inhibin beta B chain-like), the turtle *Chrysemys picta bellii* (two hits), or the bird *Apteryx australis mantelli* (two hits).

**Fig. 5.—:**
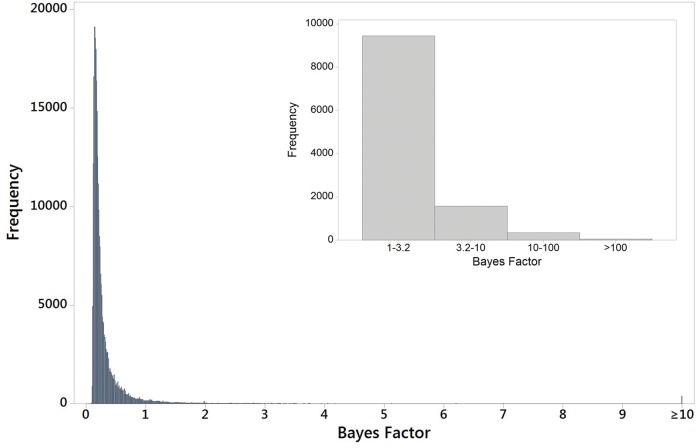
Distribution of Bayes factors from Bayenv 2 analyses of associations between SNPs and xeric/mesic habitats. The main histogram shows the distribution of Bayes factors obtained from Bayenv 2 analyses of 276,483 SNPs. The final histogram bin contains the 395 SNPs with Bayes factors ranging from 10 to 3.3 × 10^48^ (see Results). The insert histogram excludes Bayes factors <1, allowing clearer visualization of the distribution relative to cut-off points that are often used in the interpretation of Bayes factors (Kass and Raftery 1995).

For the Bayenv 2 analyses of SNP-environment correlations, the upper 4.1% of SNPS (11,417) had Bayes factors ≥1, whereas 395 SNPs (0.14%) had Bayes factors ≥10 and were therefore quite extreme relative to other SNPs (fig. 5). (Multivariate analyses of these SNPs to examine geographical patterns were not carried out as the Bayenv 2 analyses established the habitat-correlated pattern.) We compared the 103 candidate SNPs associated with selection with the 395 habitat-correlated SNPs and found that approximately one-third (36) were included in the latter group.

## Discussion

Genomic analyses of morphologically differentiated northern and southern populations provide some support for both of the non-mutually exclusive hypotheses that could explain the within-island evolution of *G. galloti*, that is, we detected the effects of both environment-correlated natural selection as well as divergence in allopatry associated with the volcanic history of Tenerife. A substantial influence by both processes may have led to high levels of diversity and explain why we detected a high SNP density across the genome, relative to other studies that have used similar paired-end RAD-seq analyses (Ogden et al. 2013; Senn et al. 2013; Pascoal et al. 2014).

Genome sizes in other distantly-related squamata that have been sequenced are 1.4–1.8 Gbp (Alföldi et al. 2011; Castoe et al. 2011; Song et al. 2015), which suggests that RAD-seq analyses covered ∼1.1–1.5% of the *G. galloti* genome. Identification of outliers from *F*_ST_ statistics provides evidence of positive selection at a small proportion of the genome (only 0.4% of SNPs). Obviously some of these sites will be in linkage disequilibrium with sites under selection rather than directly under selection. However, the proportion is considerably lower than the 5–10% of outlying loci reported by many other studies of selection across the genome (Nosil et al. 2009). While this could be due to only a relatively small amount of the genome being under selection, high levels of gene flow favoured by the small geographic scale could also be important. Two lines of evidence support the hypothesis of divergent selection between north-facing and south-facing slopes. First, SNPs that were candidates for selection when analyzed without regard to habitat type provided clear discrimination of individuals from north-facing and south-facing populations when summarised using DFA (fig. 2). Given the strong structuring between the described habitat types, we rule out the hypothesis that they are simply the outliers expected by chance alone in such a large sample of SNPs. Second, tests that explicitly examined divergent selection between north- and south-facing locations revealed significant effects at a similarly small proportion of SNPs (0.14%). Also, there was some overlap between the environment-correlated SNPs and those inferred to be candidates for positive selection. Given that the primary pattern of morphological variation is associated with location on north-/south-facing slopes (Thorpe and Brown 1989a, 1989b, 1991) our genomic findings are consistent with the prediction that these morphological differences originated from ecology-related differences in natural selection between these areas.

A consequence of divergent selection is a reduction in effective gene flow between habitats (Feder et al. 2012). This should influence only genomic sites affected directly by selection or through linkage disequilibrium, that is, only a very small proportion of the genome in *G. galloti*. A previous microsatellite study detected a reduction in gene flow across the xeric/mesic ecotone (Thorpe and Richard 2001) which would be expected under cross-genomic divergence. Hence, there is a disparity between the two studies which we cannot currently explain: we found localised effects of divergent selection on the genome while Thorpe and Richard (2001) seemed to detect a genome-wide effect. This difference is quite significant given that a key question in ecological speciation is how reduced effective gene flow at a small number of sites leads to divergence at other sites (Feder et al. 2012).

The genomic analyses also support previous findings that populations have been subject to historical isolation, as previously inferred from mtDNA analyses (Thorpe et al. 1996; Brown et al. 2006). Specifically, in terms of their generalised genomic characteristics, individuals from population 5 in the extreme NE of Tenerife are very divergent from other populations and contain the greatest number of private alleles. The population is also very similar to the other northern populations when only SNPs under selection are analyzed which suggests that divergence at neutral SNPs underpin its general genomic divergence. An explanation of drift during population isolation is therefore favored. In addition to our findings, many previous mtDNA studies have inferred historical isolation of NE populations of several different Tenerife species (discussed below). It is important to note that the population is located within the ancient Anaga region of Tenerife which was probably formed as a peripheral shield volcano around 4–5 Ma (Guillou et al. 2004; Carracedo et al. 2011). Previous studies have detected divergent mtDNA lineages within the Anaga region in all three widespread lizards on Tenerife, as well as other groups such as insects (Juan et al. 2000; Moya et al. 2004). Most notably, *Chalcides viridanus* from precisely the same locality represented one of only two Anaga skink populations that contained a divergent mtDNA lineage (Brown et al. 2000). The sampled population lies within an area of Anaga that underwent a flank collapse over 4 Ma (Walter et al. 2005), but this almost certainly predates the level of divergence detected: mtDNA studies have previously suggested colonization of Tenerife over 4 Ma (Cox et al. 2010) with intraspecific divergence around 0.8 Ma (Brown et al. 2006). Hence, it is unclear why this population should be divergent while other populations on the ancient Anaga shield volcano are not. The *G. galloti* mtDNA lineage that is associated with Anaga has expanded quite a long way west across Tenerife and is now found in populations along much of the north coast (reaching approximately site 7 in this study) and extends down the SE coast (beyond site 2 in this study) (Thorpe et al. 1996; Brown et al. 2006). It is possible that this mtDNA lineage and the cross-genome divergence detected in population 5 originated during the same period of historical allopatry. Subsequently, the Anaga mtDNA alleles showed greater migration out of the region than the corresponding nuclear alleles. Discordance between geographical patterning of mtDNA and nuclear DNA is a widespread phenomenon (Toews and Brelsford 2012), but can be difficult to explain. Here, the appearance of novel advantageous mtDNA alleles in the Anaga population could explain a rapid expansion out of this region.

Although we have focused on the primary ecological differences, between the north- and south-facing slopes, this is inevitably a simplification. In fact, analyses of SNPs that appeared to be under selection revealed three geographical groupings, with the southern slopes divided into eastern and southern/western areas. The sampled areas in the latter have slightly lower annual rainfall than the former, with the south/south–west being the most arid areas of the entire island. It is also interesting that these two southern groups correspond to two different mtDNA lineages (Thorpe et al. 1996). Tenerife is ecologically very diverse and although we have focused on the north/south differences because they are correlated with *G. galloti* morphology, other aspects of the environmental variation could also mediate differences in selection. In particular, the huge differences in altitude are also likely to be important. We intend to address this in future.

RAD-seq has allowed new insights into the contribution of vicariance and selection on genetic diversity. A limitation of RAD-seq without a reference genome is that robust inferences cannot be made about proximities/functions of RAD sequences of interest, such as those under selection. Nevertheless, it is still interesting to note how variation in an ostensibly small proportion of the genome appears to underlie the substantial morphological variation between populations. Future studies will indicate whether these lie within a specific genomic region or not, as implicated in ecological speciation of other species, for example, Turner et al. (2005) and Malinsky et al. (2015).

Possible mechanisms by which habitat differences mediate different selection regimes have been discussed previously (Thorpe and Brown 1989a). As for other Canary Island species (Brown et al. 1991; Suárez et al. 2014) selection pressures on the color pattern are interpreted in terms of different strategies linked to predation from birds such as *Falco tinnunculus*. The most obvious N/S differences are observed in the color patterns of male *G. galloti*, which appears to contain both sexual and anti-predator components. A more cryptic strategy appears to have evolved in the mesic more closed habitats in the north of the island, characterised by disruptive yellow bars on the dorsum and the relocation of sexual markings so that they are less visible from above. More conspicuous sexual advertisement is found in warmer open/arid southern habitats, in which crypsis is less effective, but higher body temperatures might facilitate higher levels of vigilance and locomotor performance. In other words, an alternative anti-predator strategy is favored. While predation-mediated ecological divergence of traits involving crypsis is well-documented in some insects (e.g., Nosil 2004), *G. galloti* could represent an important model of ecological divergence of vertebrate color patterns.

In summary, our genomic analyses indicate that both ecological divergence and ancient allopatry have combined to shape present-day genetic variation. However, the major phenotypic differences seem to be determined by habitat differences, suggesting that differential patterns of selection are driving divergence and potential speciation, rather than historical population isolation.
